# 

**Published:** 1892-09

**Authors:** 


### CONTENTS:

ORIGINAL ARTICLES: PAGE

Notes on Parasites. No. 2. By C. W. Stiles, Ph. D. .... 517
The Etiology of Southern Cattle Plague—Texas Fever. By Frank S. Billings... 526
Review of Recent Publications in Medical Zoology. By. C. W. Stiles, Ph. D.... 557
Two Cases of Albumen-Urea in Horses. By A. W. Clement, V. S.... 562

EDITORIAL:

United States Veterinary Medical Association .... 564
New Jersey State Committee of Arrangements.... 564
Applicants for Membership in the United States Veterinary Medical Association, 566

COMMUNICATIONS:

Springhalt. By Williamson Bryden.... 569

SOCIETY PROCEEDINGS:

New York State Veterinary Medical Society.... 571

